# Low-Frequency Photoparoxysmal Responses in a Patient With MELAS

**DOI:** 10.31083/RN40724

**Published:** 2025-12-25

**Authors:** Yung-Hsiu Lu, Dau-Ming Niu, Wei-Sheng Lin

**Affiliations:** ^1^Department of Pediatrics, Taipei Veterans General Hospital, 11217 Taipei, Taiwan, ROC; ^2^Institute of Clinical Medicine, National Yang Ming Chiao Tung University, 112304 Taipei, Taiwan, ROC; ^3^School of Medicine, National Yang Ming Chiao Tung University, 112304 Taipei, Taiwan, ROC

A 16-year-old female received an electroencephalography (EEG) as a follow-up examination for her epilepsy. She has been diagnosed with mitochondrial encephalomyopathy with lactic acidosis and stroke-like episodes (MELAS) at the age of 13, when she had suffered from recurrent stroke-like episodes involving right and left posterior quadrants of the cerebrum respectively during a 2-month period. Molecular testing revealed heteroplasmic (66%) m.3243A>G mutation in her mitochondrial genome extracted from peripheral blood leukocytes. The CARE checklist associated with this article can be found in the **Supplementary Material**.

She has been treated with levetiracetam, Coenzyme Q10, carnitine, and arginine. Overt seizure occurred infrequently (~1/year) in the subsequent course. Routine EEG at the last follow-up showed occasional interictal epileptiform discharges, which were similar to the findings in her previous EEG. Besides, epileptiform responses were elicited by intermittent photic stimulation (IPS) at 1 Hz (Fig. 1a), 17 Hz, and 21.5 Hz (Fig. 1c), while typical photic driving responses were seen during IPS 9 and 15 Hz (Fig. 1b).

**Fig. 1.**
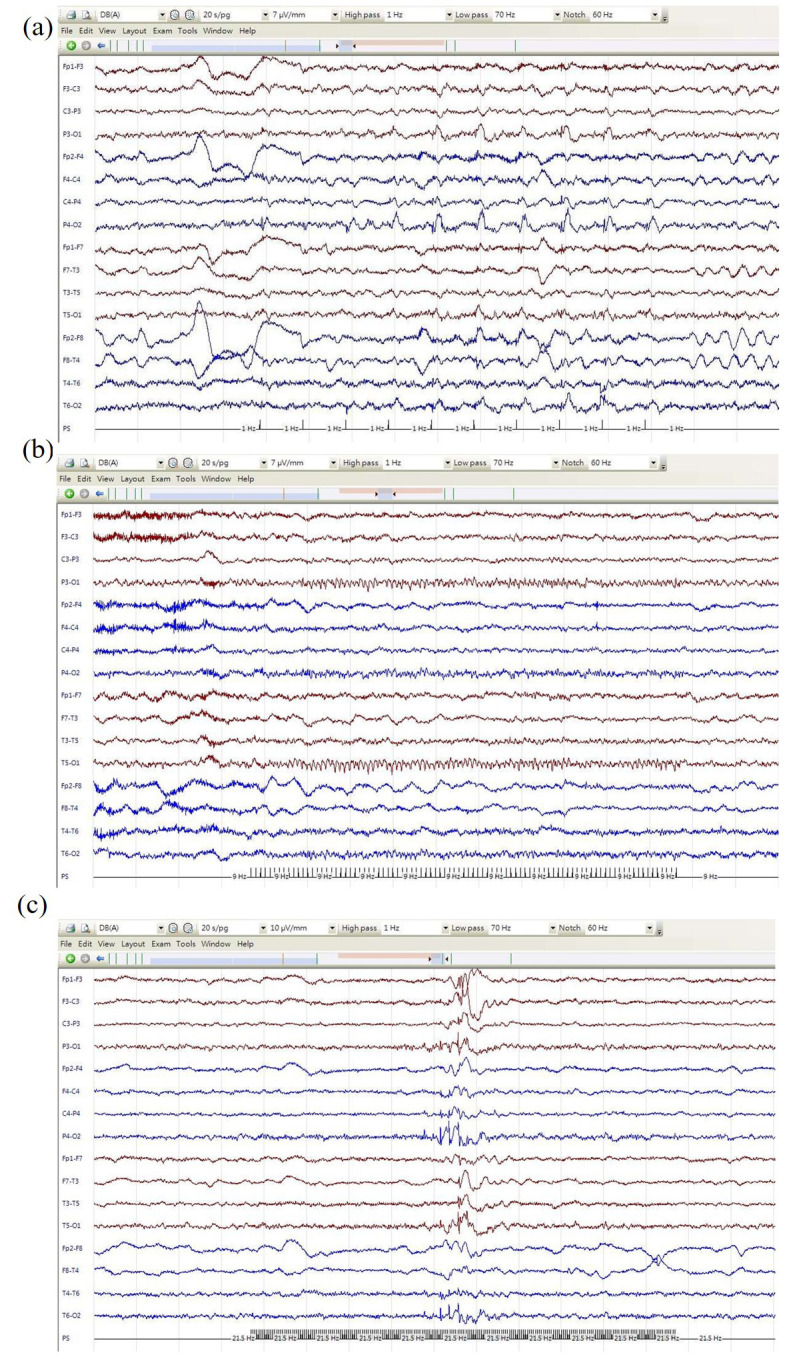
**EEG tracings during intermittent photic stimulation at (a) 1 Hz, (b) 9 Hz, and (c) 21.5 Hz**. Gain: 7 µV/mm (a,b) and 10 µV/mm (c), high-frequency filter: 70 Hz, low-frequency filter: 1 Hz. EEG, electroencephalography.

Photoparoxysmal response (PPR), a well-established marker of photosensitivity, is defined as an epileptiform electroencephalographic phenomenon provoked by IPS, which could be limited to IPS train or self-sustained [1]. PPR comprises a continuum, ranging from posteriorly dominant to generalized discharges. Low-frequency PPR, defined as PPR elicited at <5 Hz IPS, has been observed in several clinical entities belonging to the progressive myoclonus epilepsies category, including neuronal ceroid lipofuscinosis type 2 (CLN2), myoclonic epilepsy with ragged red fibers (MERRF), and Lafora disease. In addition, it has also been reported in adults with MELAS and Creutzfeldt-Jakob disease [2]. As a relatively uncommon electrophysiological phenomenon, low-frequency PPR may serve as a diagnostic clue in conjunction with the clinical contexts [3, 4].

The standard method for determining EEG photosensitivity is IPS across different frequency ranges. The frequency-response curve and its underlying brain dynamics are apparently nonlinear [5], with a lower threshold for PPRs at both low and high-frequency IPS. The rarity of low-frequency PPR suggests that a more constrained electrophysiological milieu might be required for its generation. Indeed, past EEG in this patient already showed PPRs during conventional but not low-frequency IPS. Furthermore, our patient exhibited different patterns of PPRs to high-frequency (21.5 Hz) versus low-frequency (1 Hz) IPS. The former took the form of short-lasting (~1 second) 4–5 Hz bilateral asymmetric spike-wave discharges (Fig. 1c), which were more akin to her usual interictal epileptiform activities. In contrast, the low-frequency PPRs exhibited one-to-one correspondence and time-locked to visual flashes (Fig. 1a), which might suggest the possibility of giant visual evoked potential (VEP). Nonetheless, the spike-and-wave morphology and the negative polarity of the main component of the discharges may help distinguish PPR from VEP in this case. We speculate that the low-frequency PPRs in this patient might be primarily a reflection of occipital cortex hyperexcitability [6], whereas higher-frequency IPS activated more extensive networks including preexisting epileptogenic circuits [7]. MELAS typically affects the posterior cerebral cortex, which might explain the EEG photosensitivity observed across a broader frequency range. It is important to educate patients and caregivers that photosensitive seizures may be provoked by bright, flickering visual stimuli as well as high-contrast patterns; therefore, practical preventive measures include reducing screen brightness and contrast, using flicker-free displays, and maintaining a viewing distance of at least two meters from the screen [1].

The connection between mitochondrial dysfunction and epileptic seizures is well established [8, 9]. Possible mechanisms include impaired adenosine triphosphate (ATP) production, dysregulated calcium homeostasis, and lactate accumulation, all potentially leading to altered neuronal excitability and lowered seizure threshold [9, 10]. The EEG findings in mitochondrial epilepsies are often variable and nonspecific, perhaps with the exception that rhythmic high-amplitude delta with superimposed (poly)spikes (RHADS) pattern is considered relatively specific for polymerase gamma-1 (POLG1)-related Alpers-Huttenlocher syndrome [11]. Based on this case and existing literature, low-frequency PPR appears far less common than conventional PPR, and when present in the appropriate clinical context, may serve as another relatively specific diagnostic clue for mitochondrial encephalopathy.

In conclusion, the presence of low-frequency PPR may serve as a valuable electrophysiological biomarker for MELAS and should be specifically sought in relevant clinical contexts. The exact neurobiological mechanisms underlying the frequency-specific modulation of photic evoked cortical responses remain incompletely understood and deserve further research [1].

## Availability of Data and Materials

The data that support the findings of this study are available from the corresponding author upon reasonable request.

## Supplementary Material

Supplementary material associated with this article can be found, in the online version, at https://doi.org/10.31083/RN40724.
